# Photobiomodulation (blue and green light) encourages osteoblastic-differentiation of human adipose-derived stem cells: role of intracellular calcium and light-gated ion channels

**DOI:** 10.1038/srep33719

**Published:** 2016-09-21

**Authors:** Yuguang Wang, Ying-Ying Huang, Yong Wang, Peijun Lyu, Michael R. Hamblin

**Affiliations:** 1 Center of Digital Dentistry, Peking University School and Hospital of Stomatology, Beijing, China; 2 National Engineering Laboratory for Digital and Material Technology of Stomatology, Beijing, China; 3 Wellman Center for Photomedicine, Massachusetts General Hospital, Boston, MA, 02114, USA; 4 Department of Dermatology, Harvard Medical School, Boston, MA, 02115, USA; 5 Harvard-MIT Division of Health Sciences and Technology, Cambridge, MA, 02139, USA

## Abstract

Human adipose-derived stem cells (hASCs) have the potential to differentiate into several different cell types including osteoblasts. Photobiomodulation (PBM) or low level laser therapy (LLLT) using red or near-infrared wavelengths has been reported to have effects on both proliferation and osteogenic differentiation of stem cells. We examined the effects of delivering four different wavelengths (420 nm, 540 nm, 660 nm, 810 nm) at the same dose (3 J/cm^2^) five times (every two days) on hASCs cultured in osteogenic medium over three weeks. We measured expression of the following transcription factors by RT-PCR: RUNX2, osterix, and the osteoblast protein, osteocalcin. The 420 nm and 540 nm wavelengths were more effective in stimulating osteoblast differentiation compared to 660 nm and 810 nm. Intracellular calcium was higher after 420 nm and 540 nm, and could be inhibited by capsazepine and SKF96365, which also inhibited osteogenic differentiation. We hypothesize that activation of light-gated calcium ion channels by blue and green light could explain our results.

Human adipose-derived stem cells (hASCs) have emerged as a popular and versatile tool in the field of regenerative medicine^1^. Adipose tissue is usually isolated in the form of fat removed during liposuction procedures. This tissue represents an abundant and accessible source of adult stem cells that can be purified from the lipoaspirate, with the ability to differentiate along multiple lineage pathways^2^. hASCs have been shown to be very similar (in terms of markers expressed on their surface and in their differentiation potential) to bone marrow-derived mesenchymal stem cells (BMDMSC)^3^.

Many surgical and orthopedic procedures require the reconstruction of significant defects in bone, which are beyond the already excellent capacity of natural bone to heal, because they are too large^4^. Autologous bone graft which is usually harvested from the iliac crest, is considered to be the gold standard material for bone regeneration in orthopedic surgery^5^. However the autologous bone graft procedure has limitations including donor site morbidity, limited amounts, and a requirement for a second surgical procedure.

To overcome these limitations, researchers have proposed the use of HADSC to provide a source of cells that can differentiate and proliferate into osteogenic cells (osteoblasts) under the influence of the appropriate molecular signals^6^. These signals can be partly provided by an appropriate scaffold with the correct properties: a three-dimensional structure, a composition consisting of polymers (e.g. poly-lactic-co-glycolic acid), proteins (e.g. collagen) and minerals (e.g. hydroxyapatite)^7^. In addition to the correct scaffold exogenous growth factors are often added into the mix. These growth factors may contain bone morphogenic proteins (BMPs) which are members of the TGF-α superfamily, as well as osteopontin, fibronectin tenascin, and bone sialoprotein^8^.

Nevertheless, despite much information that is known about how to induce these hASCs to differentiate into osteoblasts^9^,^10^, it is always desirable to find additional inexpensive and harmless interventions that could accelerate the process, and increase the yield of the desired bone cells. Such a method which certainly qualifies as inexpensive and harmless is photobiomodulation (PBM), also known as low level laser therapy (LLLT)^11^. PBM has been used for several years as a specific way of stimulating various types of stem cells to proliferate and differentiate^12^. Several studies have examined this process *in vitro*^13^,^14^,^15^,^16^,^17^,^18^,^19^,^20^,^21^,^22^. There have been some *in vivo* studies that have for instance tried seeding spheroids made of BMDMSC onto excisional wounds in mice and irradiate them or not with light^23^,^24^. Other studies have used a laser to irradiate the tibias of mice with the aim of mobilizing stem cells from the bone marrow that could then migrate and repair a heart attack^25^,^26^, or reverse ischemic kidney injury^27^.

Despite many publications shining light on hASCs and BMDMSC *in vitro*, it is still rather unclear what are the optimum wavelengths for this process and what are the most appropriate doses of light. The wavelengths that are generally used in PBM, to some extent depend on what specific chromophores inside the cells are proposed to be targeted. For the best-established cellular chromophore within the mitochondria, namely cytochrome c oxidase, it is reasonably well accepted that either red light (630 nm–670 nm) or near-infrared light (780 nm–940 nm) will have positive effects, provided the dose employed is kept within the stimulatory range (a few J/cm^2^). However, evidence is emerging that ion channels within cells can also respond to light, but the optimum wavelengths for this effect are unknown. In the present study we compared four different wavelengths (blue 420 nm, green 540 nm, red 660 nm and near infrared 810 nm) all delivered at the same fluence (3 J/cm^2^) on the osteogenic differentiation of hASCs *in vitro*.

## Material and Methods

### Cell culture

Human adipose-derived stem cells (hASCs) were purchased from ScienCell Company (San Diego, CA, USA). All materials were purchased from Sigma-Aldrich (St. Louis, MO, USA) unless noted otherwise. Fetal bovine serum (FBS) was purchased from Atlanta Biologicals (Flowery Branch, GA, USA). Proliferation medium (PM) is composed of Dulbecco’s modified Eagle medium (DMEM, Gibco BRL, Grand Island, NJ, USA) containing 10% fetal bovine serum, 100 IU/ml penicillin/streptomycin. Osteogenic differentiation medium (OM) is composed of high glucose Dulbecco’s modified Eagle medium (DMEM) containing 10% fetal bovine serum, 100 IU/ml penicillin/streptomycin, 100 nM dexamethasone, 0.2 mM ascorbic acid, and 10 mM β-glycerophosphate.

### Photobiomodulation and pharmacological compounds

The cells were irradiated by 4 different wavelengths of photobiomodulation (420, 540, 660, 810 nm) at the dose of (3 J/cm^2^) five times (every two days) on hASCs cultured in osteogenic medium for three weeks. The different light sources are listed in Table 1. The chemicals were added into the culture medium 10 min before photobiomodulation. Table 2 shows the time course of photobiomodulation on cells cultured in OM and gene expression measurement.

Capsazepine (CPZ) is a selective inhibitor of transient receptor potential vanilloid 1 (TRPV1) channel, and SKF96365 (SKF) is a non-selective transient receptor potential canonical (TRPC) inhibitor. CPZ and SKF were dissolved in DMSO at a concentration of 10 mM and a final concentration of 5 uM was used for the experiments.

### RNA Extraction, Reverse Transcription, and Quantitative RT-PCR

In order to evaluate the effects of different wavelengths of photobiomodulation and TRP channel inhibitors on osteogenic markers, quantitative PCR was performed. Total cellular RNAs were isolated with RNeasy Mini Kit (QIAGEN, Valencia, CA) and used for High-Capacity RNA-to-cDNA™ Kit System (Applied Biosystems, Foster City, CA). Quantification of all gene transcripts was performed by real-time polymerase chain reaction (RT-PCR) using a SYBR Green kit (Roche Diagnostics Ltd, Lewes, UK). GAPDH was used as an internal control. The primers used are listed in Table 3.

### Sulforhodamine B colorimetric assay

In order to find a suitable drug concentration of the TRP inhibitor, we measured the cell proliferation by Sulforhodamine B colorimetric assay which measures amount of cellular protein and does not rely on mitochondrial activity. Briefly, cells were seeded at 3,000 per well in a 96-well plate and culture for one day. After stimulating by drugs, cells were fixed by 10% (wt/vol) trichloroacetic acid for 30 min and stained by 0.057% SRB solution for 30 min. After washing by 1% (vol/vol) acetic acid, the samples were dissolved in 10 nM Tris base solution, and OD was measured at 510 nm.

### Intracellular calcium assay

To monitor the changes in the intracellular calcium concentration, hASCs in osteogenic medium were pretreated with 1 μM Fluo-4 AM for 1 hour before photobiomodulation. Then different wavelengths of photobiomodulation were applied and confocal images were taken immediately.

### Alizarin red S (AR-S) staining and mineralization assays

To detect osteogenic differentiation, the hASCs were seeded in 6-well plates and cultured with osteogenic medium (OM) for 14 or 21 days then used for mineralization testing. For qualitative testing, plates were washed three times with PBS, hASCs were fixed with 95% ethanol, then stained with 0.5% alizarin red stain for one hour. After staining, the cells were washed with distilled deionized water. Positive stained cells were then detected with an optical microscope. For quantitative detection, the stained samples were solubilized by 100 mM cetylpyridinium chloride to dissolve the calcium-bound AR-S and then the solution was transferred to 96-well plate, 100 microliters per well, and the absorbance was measured at 562 nm. The experiment was repeated three times.

### Statistical analysis

All data were performed in triplicate with n = 6/8 for each sample. Software SPSS 19.0 (SPSS Inc., Chicago, IL, USA) was used to perform one-way ANOVA with Tukey’s post-hoc test to evaluate the statistical significance of all results (p < 0.05). For multiple comparisons, Bonferroni was used in all the experiments. The 2^delta delta Ct method was used in relative gene expression studies.

## Results

### RUNX2, OCN, OSX expression in culture after 420 nm, 540 nm, 660 nm and 810 nm photobiomodulation

**Figure 1:**
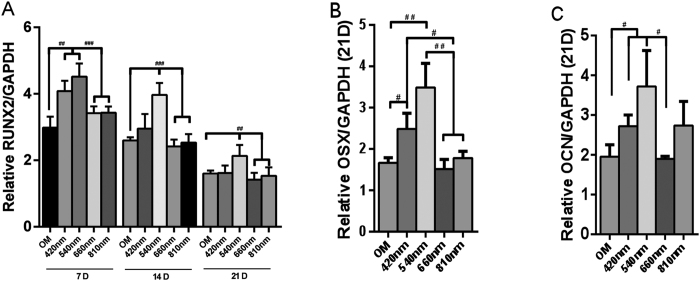
Quantitative evaluation of mRNA levels via real-time PCR of RUNX2 (**A**), OSX (**B**) and OCN (**C**) after 4 different wavelengths (420, 540, 660 and 810 nm) PBM. Data are expressed as mean ± SD. Experiments were carried out using two dishes each in three experiments (n = 6). ^#^p < 0.05, ^##^p < 0.01, ^###^p < 0.001.

An analysis for evaluating the mRNA levels of RUNX2, OCN, and OSX was performed with or without photobiomodulation (PBM). The expression of RUNX2 demonstrated that hASCs differentiate into osteoblasts in culture. For RUNX2 gene expression, we examined mRNA level at 7 days, 14 days and 21 days. PBM was used every two days, so for 7 days group PBM was used 4 times, while 14 days and 21 days groups we used PBM 5 times. We found that the RUNX2 level of the green light group at all three time points were higher than red, near infrared and OM groups. The blue light group was higher than red light, near-infrared and OM group at 7 days (Fig. 1A). For OSX gene expression, the green and blue PBM groups had better effects than the red, near infrared and OM groups at 21 days (Fig. 1B). For OSX gene expression, at 21 days, we found that the green light PBM group was better than the red and OM groups, and the blue light group was better than OM group (Fig. 1C).

### The activation of 420 nm and 540 nm to promote osteogenic differentiation could be abrogated by TRPV1 and TRPC channel inhibitors

**Figure 2:**
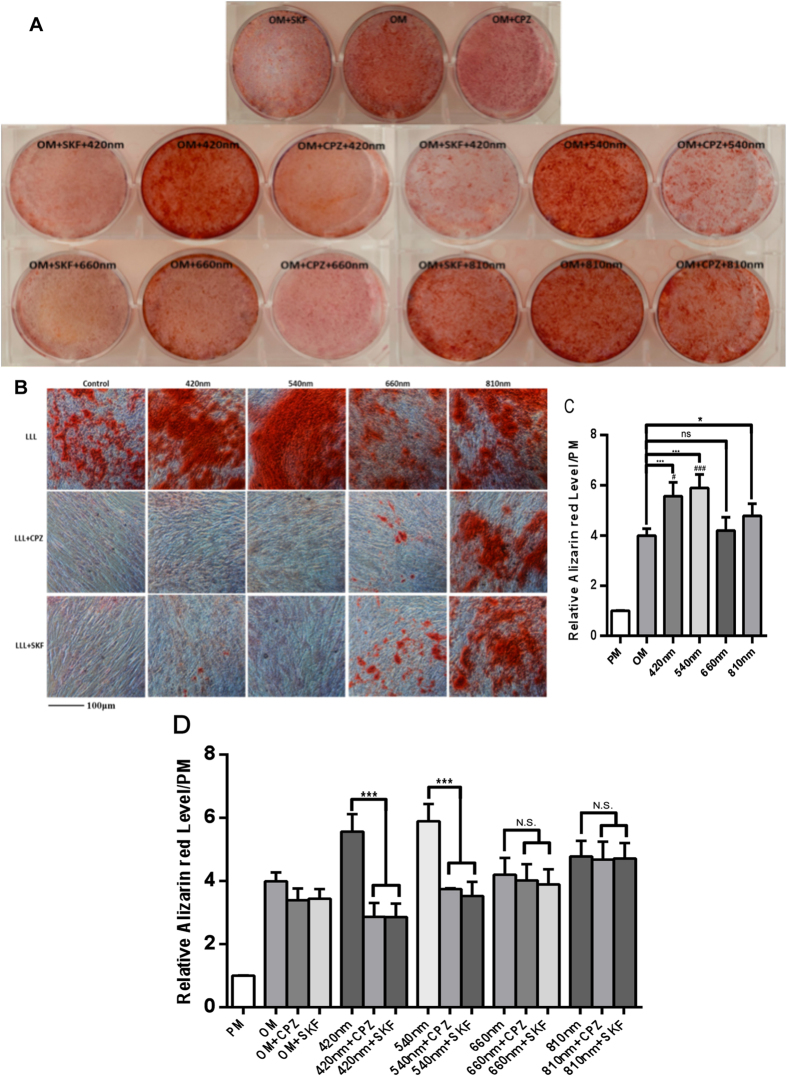
(**A**) Alizarin red stain was added into cell cultures in osteogenic medium after photobiomodulation at a dose of 3 J/cm^2^ five times (every two days) with or without CPZ or SKF pretreatment. The alizarin red staining was measured after 21 days to determine the level of mineralization. Pre-incubation with CPZ (5 μM) and SKF (5 μM) for 10 minutes before photobiomodulation reduced the effect of photobiomodulation in 420 nm and 540 nm groups, to a lesser extent in the 660 nm group, but not in the 810 nm group. (**B**) Images of alizarin red staining taken by microscope. A higher intensity of alizarin red after 420 nm and 540 nm groups, while the intensity of 420 nm and 540 nm +CPZ/SKF groups was similar to the control group. (**C**,**D**) Quantitative evaluation of calcium deposits using Alizarin red staining. hASCs were treated or not with the TRP channel inhibitors CPZ (5 μM) and SKF (5 μM) for 10 minutes before each application of photobiomodulation. Data are expressed as mean ± SD. Experiments have been carried out for 3 times (n = 8). *,^#^P < 0.05, ***,^###^P < 0.001.

We performed Alizarin red (AR-S) staining as a mineralization assay in osteogenic medium with or without addition of TRP channel antagonists CPZ(5 μM) and SKF(5 μM) incubating for 10 minutes before photobiomodulation. There was a significant difference between OM and 420 nm, 540 nm, 810 nm groups. ***(P < 0.001) for 420 nm and 540 nm groups, and *(P < 0.05) for 810 nm group. There was no significant difference between the OM and 660 nm groups. Compared with 810 nm group, 420 nm (^#^P < 0.05) and 540 nm (^###^P < 0.001) had better effects in the ARS assay (Fig. 2A–C). The increase in the mineralization level in response to 420 nm and 540 nm groups was abrogated by the TRP channel antagonists CPZ and SKF (Fig. 2A–D). These results imply that TRP calcium channels play a role in blue and green light-enhancement of osteoblast differentiation. The AR staining after red light (660 nm) was partially abrogated by the TRP inhibitors. NIR light-mediated enhancement of osteogenic differentiation was not abrogated by TRP inhibitors, and therefore appears to occur via a different mechanism.

### 420 nm and 540 nm photobiomodulation increase osteogenic relative gene expression through TRP/calcium signaling pathway

**Figure 3:**
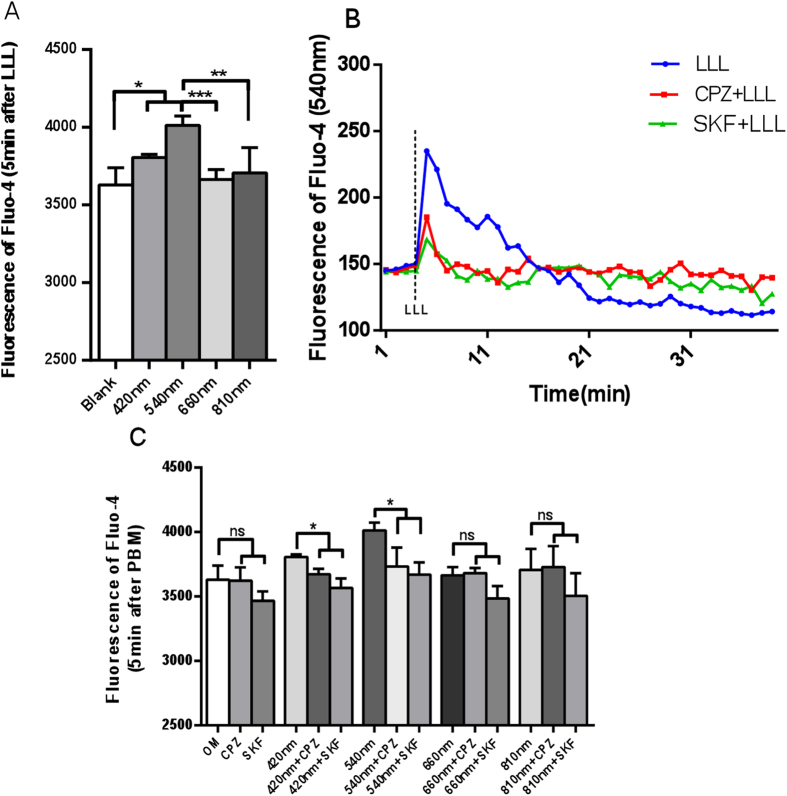
CPZ and SKF blocked the increase of intracellular calcium in hASCs cultured in OM caused by 420 nm or 540 nm. (**A**) Effects of four different wavelengths on intracellular calcium measured immediately. (**B**) Time course of intracellular calcium after 540 nm with or without CPZ or SKF. (**C**) Quantitative analysis for intracellular calcium with or without CPZ (5 μM) or SKF (5 μM) pretreated before photobiomodulation using all four wavelengths.

The expression of osteogenic genes Runx2, OCN and OSX could be regulated by intracellular calcium, which could in turn be elevated by blue and green light. In order to investigate whether intracellular calcium was elevated by blue and green light, hASCs were pretreated with CPZ (5 μM) or SKF (5 μM) 10 min before photobiomodulation. Fluo-4 was used as a fluorescent indicator to measure calcium levels immediately after light and RT-PCR was used to measure osteogenic gene expression after 21 days. We found that 540 nm laser irradiation at 3 J/cm^2^ gave the highest increase in intracellular calcium concentration followed by 420 nm. 660 nm and 810 nm wavelengths did not significantly increase calcium (Fig. 3A). The increase in calcium occurred within 1 min after cessation of 540 nm illumination (Fig. 3B).

The increase of intracellular calcium in response to 420 nm and 540 nm groups was abrogated by TRP channel antagonists CPZ and SKF (Fig. 3B,C). SKF also reduced calcium in hASCs in OM alone (no light) but this was not significant. In 660 nm and 810 nm groups there were no significant differences between photobiomodulation group and CPZ or SKF pre-treated groups with intracellular calcium (Fig. 3C).

**Figure 4:**
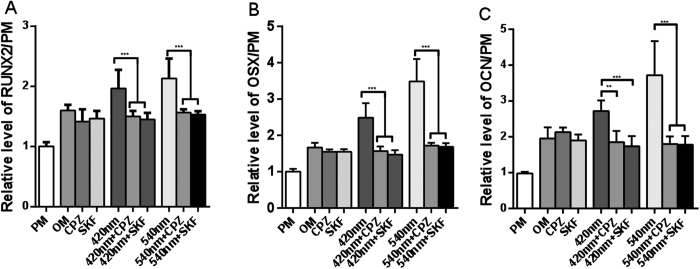
Effects of TRP inhibitors on osteogenic gene expression stimulated by 420 nm or 540 nm photobiomodulation. (**A**) Quantitative analysis for gene expression level of RUNX2. The data of RUNX2 expression are shown at day 21; data at days 7 and 14 are not shown. (**B**) Quantitative analysis for gene expression level of OSX at day 21. (**C**) Quantitative analysis for gene expression level of OCN at day 21. Data represent means ± SD of the number of determinations (n = 4 or 6, *P <0 .05, **P < 0.01, ***P < 0.001).

Incubation with CPZ (5  μM) or SKF (5 μM) before each individual application of 420 nm and 540 nm photobiomodulation delivered 5 times over 21 days, significantly decreased RUNX2, OSX, and OCN expression levels as compared to the control group (OM alone) (Fig. 4A–C). In the 660 nm and 810 nm groups the relative gene expression levels showed no differences in the CPZ or SKF pretreated groups compared to OM alone (Data not shown).

## Discussion

The present study has found some interesting and surprising results related to the effects of four different wavelengths in promoting osteogenic differentiation of hASCs. Other previous studies using photobiomodulation for stem cell differentiation have mainly used red light (and occasionally NIR light) to promote osteogenic differentiation of various kinds of stem cells including hASCs. Abramovitch-Gottlib *et al*. used a HeNe laser (632.8 nm) to promote osteogenic differentiation of a mouse MSC cell line growing on a three-dimensional (3D) coralline biomatrix^13^. Peng *et al*. used red LEDs (620 nm) to promote osteogenic differentiation of primary rat BMDMSC and measured up-regulation of various osteoblast related genes^28^. Li *et al*. performed similar studies on primary rat BMDMSC using a 630 nm LED array^29^. Soleimani and coworkers^22^ used the NIR wavelength (810 nm laser) to promote osteogenic differentiation of hASCs. All these previous reports used comparable fluences (a few J/cm^2^), and often repeated the light irradiation several times over the entire course of the experiment.

Since we originally expected the red (660 nm) and NIR (810 nm) wavelengths to have the most pronounced effect on stimulating the osteogenic differentiation of hASCs, we were somewhat surprised to find that the blue (420 nm) and the green (540 nm) wavelengths in fact had much better effects on this differentiation process, when compared to the red and NIR wavelengths. It is reasonably well established^11^ that red and NIR light activates cytochrome c oxidase (CCO, unit 4 in the mitochondrial respiratory chain). This CCO activation is proposed to occur by displacing inhibitory nitric oxide^30^, and the consequent increased activity of CCO increases mitochondrial membrane potential thus allowing the mitochondria to produce more ATP. The particular effect of red and NIR light in promoting stem cell differentiation, is proposed to be due to shifting the metabolic profile from glycolysis to oxidative phosphorylation due to the increased mitochondrial number and activity induced by the light exposure. It is known that this metabolic switch (glycolysis to oxidative phosphorylation) is a key factor in stem cell osteogenic differentiation^31^. Moreover photobiomodulation can also cause a brief production of reactive oxygen species (ROS)^32^, and ROS production has also been shown to be involved in stem cell differentiation^33^.

RUNX-2 is now recognized as one of the most important osteogenic differentiation transcription factor. Osteocalcin (OCN) is non-collagenous protein which found specific in bone, and is also considered to be a marker of osteoblast differentiation during bone metabolism process. Osterix (OSX) is an important transcription factor in the end stage of osteoblast. differentiation which determines the expression of a variety of osteoblast markers. And OSX has essential effects in bone formation which maybe a downstream transcription factor of RUNX-2.

The ability of TRP channel inhibitors such as CPZ and SKF to abrogate the response of hASCs to blue and green light suggested that light-gated ion channels (as opposed to mitochondrial stimulation) may be involved in this response.

In recent years there has been an enormous amount of interest in light-gated ion channels^34^. Light-gated channelrhodopsin cation channels (originally isolated from chlorophyte algae) have transformed neuroscience research through their use as membrane-depolarizing optogenetic tools for targeted photoactivation of the firing of neurons^35^. A recent report described the isolation of light-gated anion channels with faster kinetics than channelrhodopsin, triggered at less than one-thousandth of the light intensity^36^. The chromophore in channelrhodopsin relies on *cis-trans* isomerizarion of a retinaldehyde molecule producing reversible alteration of the tertiary protein structure. The action spectra of the family of chennelrhodopsins mainly shows peaks in the blue-green spectral region, although variants are now known with peaks ranging all the way from 436 nm to 587 nm^37^.

The superfamily of ion channels known as transient receptor potential (TRP) channels was originally discovered as a light-gated calcium channel in a Drosophila mutant that was defective in visual transduction^38^. TRPs are non-selective cation channels with six transmembrane domains, and have now expanded into a huge superfamily of seven different sub-classes based on sequence homology^39^, members of which are present in almost all known life forms^40^. The vanilloid TRP sub-class (TRPV) was identified as including the receptor (TRPV1) specific for capsaicin (active ingredient in hot chilli peppers) originally found in the dorsal root ganglia^41^. TRPVs have now been shown to have a multitude of biological functions, including perception of pain, pressure and heat, and are involved in several brain functions^42^.

Wang *et al*.^43^ studied activation of the TRPV1 channel that had been exogenously expressed in Xenopus oocytes by red (637 nm) and green (532 nm) laser light. They found (in agreement with their previous study in mast cells^44^) that red laser activated TRPV1, but also discovered that green laser produced an even more pronounced activation. Laser activation in mast cells was abrogated by SKF and ruthenium red (a broad-spectrum inhibitor of mammalian ion channels). Gu et^45^ showed that green (532 nm) light activated TRPV1 expressed in Xenopus oocytes, but this activation did not occur with blue (406 nm) or with red (637 nm) light. Although TRPV channels are not yet generally accepted to be light-gated ion channels, a recent report suggests that thermosensitive TRPV1 and TRPV4 channels are expressed in the pineal photoreceptor cells of a teleost fish, where they modulate melatonin secretion *in vitro*^46^.

Melanopsin was identified as a photoreceptor molecule expressed in intrinsically photosensitive retinal ganglion cells in mammalian organisms (including humans)^47^. Melanopsin is responsible for regulating circadian rhythms^48^, and the melanopsin chromophore also relies on isomerization of 11-*cis* retinal (with a peak at 479 nm) producing a rise in intracellular calcium^49^. Melanopsin has been targeted by various therapeutic devices that use bright white or blue light shone in the face to treat jet-lag, seasonal affective disorder, insomnia and depression^50^,^51^,^52^. In 1998 Campbell and Murphy^53^ proposed that bright light delivered to the back of the knees could have similar effects on circadian rhythms, as when shone in the eyes, but this study was later challenged^54^.

The fact that SKF was more effective than CPZ in abrogating the effects of green and blue light in our hASCs differentiation system, suggests that TRPV1 may not be the main (or indeed the only) light gated ion channel operating in these hASCs.

Further work is needed to investigate in more detail the mechanism of action of different wavelengths of photobiomodulation on various different types of ion channels. So far this response has only been shown to naturally occur in mast cells and now in hASCs. How many other cell types also respond in this manner to blue or green light? It will no doubt be pointed out that since the transmission of blue and green light by tissue is very limited, therapeutic applications of blue and green light will be doomed to failure. However the Philips Company has introduced a blue light patch called “BlueTouch” for relief of back pain (https://www.philips.co.uk/c-p/PR3082_00/bluetouch-bluetouch-pain-relief-patch/overview) although we cannot trace any peer-reviewed publications supporting its efficacy. Could this device be operating via activation of light-gated ion channels? Moreover many therapeutic applications of stem cells require expansion and differentiation protocols to be carried out *in vitro* before introduction into the site of injury or disease, and it may be possible to use different wavelengths of light for these two different purposes. In other words, use red/NIR light for expansion and proliferation of stem cells, and use blue/green light for differentiation into progenitor cells.

## Additional Information

**How to cite this article**: Wang, Y. *et al*. Photobiomodulation (blue and green light) encourages osteoblastic-differentiation of human adipose-derived stem cells: role of intracellular calcium and light-gated ion channels. *Sci. Rep.* **6**, 33719; doi: 10.1038/srep33719 (2016).

## Figures and Tables

**Table 1:** Light sources and parameters.

Wavelength	410–430 nm	525–555 nm	660 nm	810 nm
Type	LED array	Filtered lamp	Diode laser	Diode laser
Manufacture	OMNILUX, CA	LumaCare™ Lamp, CA	Arroyo Instruments, LLC, CA, USA	Opto Power Corp., Tucson, AZ, USA
Models	D35PN EL 1600	Model LC-122 Medical	5305 TECSource, 5 A/12 V, 4308 LaserSource, 8 A	Model D030-MM-FCTS/B
Mode	CW	CW	CW	CW
Fluence rate (mW/cm^2^)	16	16	16	16
Fluence (J/cm^2^)	3	3	3	3
Time of irradiation (s)	188	188	188	188
Spot size (cm^2^)	4	4	4	4

The fluence rate was adjusted by changing the distance between the laser and the cell culture dish. The cell culture plates were covered with aluminum-foil, spot size was defined by the size of window in the aluminum-foil. CW, continuous-wave.

**Table 2:** Application of photobiomodulation on cells cultured in OM and gene expression time course.

Days in OM (day)	Application of PBM (time)	Gene expression
0	1	
2	2	
4	3	
6	4	
7		RUNX2
8	5	
14		RUNX2
21		RUNX2, OCN, and OSX

**Table 3:** The primers for qPCR Analysis.

	Forward primer	Reverse primer
ALP	ATGGGATGGGTGTCTCCACA	CCACGAAGGGGAACTTGTC
RUNX2	CCGCCTCAGTGATTTAGGGC	GGGTCTGTAATCTGACTCTGTCC
OCN	CACTCCTCGCCCTATTGGC	CCCTCCTGCTTGGACACAAAG
OSX	AGCAGCAGTAGCAGAAGCA	CAGCAGTCCCATAGGCATC
GAPDH	GGTCACCAGGGCTGCTTTTA	GGATCTCGCTCCTGGAAGATG
